# Long Term School Based Deworming against Soil-Transmitted Helminths Also Benefits the Untreated Adult Population: Results from a Community-Wide Cross Sectional Survey

**DOI:** 10.1155/2019/4151536

**Published:** 2019-05-02

**Authors:** Paul M. Gichuki, Gabriel Mbugua, Edwin K. Kiplelgo, Tabitha W. Irungu, Charles Mwandawiro

**Affiliations:** ^1^Eastern and Southern Africa Center for International Parasite Control (ESACIPAC), Kenya Medical Research Institute (KEMRI), Kenya; ^2^School of Health Sciences, Meru University of Science and Technology, Meru, Kenya; ^3^Center for Biotechnology Research and Development (CBRD), Kenya Medical Research Institute (KEMRI), Kenya; ^4^Center for Microbiology Research (CMR), Kenya Medical Research Institute (KEMRI), Kenya

## Abstract

**Background:**

Soil-transmitted helminths (STH) are a public health problem in Kenya. The primary control strategy for these infections is preventive chemotherapy (PC) delivered through school based deworming (SBD) programs. The World Health Organization (WHO) recommends the inclusion of other at-risk groups in the PC. The untreated groups in endemic areas have been shown to act as reservoirs for STH transmission. Few field based studies have focused on the possible benefits of SBD to the untreated groups in the community. This study sought to determine the levels of STH among all age groups in a community where SBD has been going on for more than 10 years.

**Methods:**

This was a cross sectional study where 3,292 individuals, ranging from 2 to 98 years, were enrolled. Stool samples were analyzed using duplicate Kato Katz thick smear technique for presence of STH eggs. Statistical analysis was conducted using STATA software 14.0 (Stata corporation).

**Results:**

Out of the total 3,292 stool samples analyzed, only 13 were positive for any STH. Of these, 12 were infected with* Trichuris trichiura* and one case was of hookworm. There was no* Ascaris lumbricoides *infection detected. Of the 13 STH infections, seven of the infections were of school going age (6-18 years), 5 were of preschool age (<6 years), and one was of adult age group (18>). More male (61.5%) than female were infected with STH.

**Conclusion:**

This study shows very low prevalence of STH among all age groups in Mwea, suggesting that long term SBD may also be benefitting the untreated groups in the community and thus the potential to achieve STH elimination in such endemic areas.

## 1. Introduction

Soil-transmitted helminths (STH) are a group of neglected tropical diseases (NTDs) that include Hookworm (*Necator americanus* and* Ancylostoma duodenale*), roundworm (*Ascaris lumbricoides*), and whipworm (*Trichuris trichiura*) [1]. Morbidity induced by these infections results in an estimated 5.19 million disability adjusted life years (DALYs) [2] and substantial productivity loss in endemic countries [3]. It is estimated that 1.4 billion individuals are infected with* A. lumbricoides, *1.3 billion with hookworms, and 1.0 billion with* T. trichiura *[4, 5] in nearly 166 countries globally [2]. In Kenya, STH infections are widely distributed with more than 10 million people being infected and approximately 16.6 million people being at risk [6].

The control strategies for STH in most endemic countries currently involve preventive chemotherapy (PC) delivered through school based deworming (SBD). However, in the recent past, there has been discussion on whether the emphasis of the WHO strategy should shift from morbidity control to the interruption of transmission [7, 8]. The WHO recommends four drugs for treatment of STH, albendazole (dose of 400 mg) and mebendazole (dose of 500 mg), which are widely used in programs, levamisole (dose of 2.5 mg kg^−1^), and pyrantel pamoate (dose of 10mg kg^−1^) which is less commonly used [9, 10].

The WHO goal to scale up chemotherapy for STH was endorsed in the London declaration of 2012, so that by 2020, 75% of the preschool and school going children in need will be treated regularly [1]. Recent WHO data shows that by the year 2017, 743 million people had received preventive chemotherapy for STH (188 million preschool-aged children, 410.1 million school-aged children, and 127.9 million women of reproductive age). [11]. These are the three priority groups identified by WHO for deworming through mass drug administration [12]. However in Kenya, only school going children are targeted in the SBD program [13] as they typically harbor chronic and intense infections at a time when they are undergoing physical and/or cognitive development [12, 14] and the relative ease of access to children in rural areas through schools [15].

However, reinfection after chemotherapy has been shown to commonly occur [16, 17]. This has been largely attributed to the subgroups in the larger community who usually are not covered in the SBD, including the preschool going children and the adult community [18]. These groups of people although untreated sometimes have disproportionately heavy burden of infection [19]. Another factor is the ability of the helminth eggs and larvae to survive for extended periods in the environment thus creating a source for rapid reinfection following chemotherapy [20]. Thus long term effectiveness of school based deworming in interrupting STH infection is dependent on maintenance of regular treatment [19].

In Mwea, Kirinyaga County, Central Kenya, annual SBD with albendazole covering all the primary schools in the area has been going on since the year 2004 [21]. The deworming programme was initiated through collaboration between Japan International Corporation Agency (JICA) and Kenya Medical Research Institute (KEMRI), Eastern and Southern Africa Center of International Parasite Control (ESACIPAC), and involves annual mass drug administration of antihelmintics to all school-aged children [21]. The treatment includes a single dose of 40 mg/kg of praziquantel using the tablet dose pole to determine the number of tablets [22, 23] and albendazole in a 400mg single dose. The deworming program temporally stopped for two years (2010 and 2011) and resumed in the year 2012 under the Kenya National school based deworming programme.

Before the start of the above program in the year 2004, a baseline parasitological survey on STH and schistosomiasis was conducted in a total of 86 schools in Mwea. All children in these schools were then treated annually with albendazole (400mg) and praziquantel [21]. Treatment coverage between the years 2004 and 2009 was reported at approximately 40,000 school going children [24]. During the years 2012-2013, treatment coverage for Mwea was 48,602 [25], while in 2013-2014, the coverage was at 46,999 [26]. The annual treatment of the school going children has continued in all the schools in the area over the years, except for year 2010 and 2011 when the program stopped temporally.

After the initial mass deworming, a cohort of 2300 children aged between 4 and 18 years from 5 sentinel schools were followed up at multiple points each year for four years and examined for intestinal helminths. The result of the above study indicates that at baseline, the prevalence of any STH was 19.2 % (95% CI 0.5-1.2%). Hookworm was at 16.7% (95% CI 15.2-18.2%),* Ascaris lumbricoides* was at 1.7% (95% CI 1.1-2.2%), and* Trichuris trichiura* was at 0.8% (95% CI 0.5-1.2%). The mean intensity as measured by eggs (per gram of faeces 9 epg) was at 1.647 for hookworm, 0.245 for* Ascaris lumbricoides*, and 0.053 for* Trichuris trichiura*. Among the 4 age groups analyzed in the study (3-5, 6-10, 11-15, and 16-18 years old) prevalence of hookworm was highest among the 16-19-year-old and decreased steadily to the 3-5-year-old patients [21].

Other studies previously carried out in the area have reported soil eating, hand washing, and shoe wearing as risk factors for STH infection [27]. Sanitation coverage among households in the area has been reported at 73% [28]. In the schools, a study reported that more than 50% of toilets were in poor hygienic conditions [29]. The study further notes that as at the year 2014, not much had changed in regard to sanitation facilities in the schools [29].

Adult age groups who do not have access to STH treatment have been shown to benefit from the school based deworming as a result of its impact on the overall transmission intensity within that population [30]. Treatment of preschool and school age children reduces the output of infective stages in faeces that result in the contamination of the environment in which everybody else lives [31]. Surveillances which are school based have been shown to give good estimates of the reduced morbidity in children but can lead to misleading estimates of the impact of these programs on overall transmission in the community [32]. Most of the studies that have been carried out in this area have focused on school going children [24, 33]. Very few field based studies have focused on the possible effect of the ongoing school based deworming programs on the larger community. Much of this information comes from mathematical modeling studies, whose findings do not anticipate an impact of the SBD on the larger community [24]. Field based epidemiological data on community-wide prevalence and intensity of STH especially in areas where school based deworming is ongoing is important so as to inform decision-making on the feasibility of including the larger community in the SBD programs, and also the potential benefits of the SBD to the untreated groups. The current study sought to determine the prevalence of STH among all age groups in a community where SBD has been going on for more than 10 years.

## 2. Materials and Methods

### 2.1. Study Area and Population

The study was conducted in Mwea East and West Subcounties of Kirinyaga County, Central Kenya. It is located about 100kms north east of Nairobi and has two rainy seasons, the long rains and the short rains. It has an estimated 154,220 households and a total population of 528,054 persons, with an annual growth rate of 1.5 percent. The population was projected to be 595, 379 in 2017. The majority of the population are of Kikuyu tribe [34]. Mwea East and West Subcounties are home to the giant Mwea irrigation scheme. Rice and horticultural farming are the main socioeconomic activities in the area. Several water channels crisscross the area supplying irrigation water to the farms and villages, respectively. STH was previously determined to be endemic in this area [21, 24].

### 2.2. Study Design and Sample Size

This was a cross sectional study carried out between the months of August and October 2017. A minimum sample size of 905 households was calculated using the formulae by Fishers et al [35]. From each household sampled, all members of the household present were asked to provide a stool sample.

### 2.3. Study Population and Selection Criteria

Multistage sampling was used where two locations from each of the two sub-counties were randomly selected. Mwea East Subcounty had a total of five locations including Murinduko, Tebere, Nyangati, Kangai, and Gathigiriri while Mwea West Subcounty had three locations Wamumu, Thiba, and Mutithi. Using simple random sampling, one location from each of the two subcounties was selected to participate in the study. Thus, Tebere and Thiba locations from Mwea East and West Subcounties, respectively, were selected.

From the locations selected, a list of all villages in each location was obtained. Thiba location had a total of eight villages including Mbui Njeru, Maendeleo, Gakungu, Rurumi, Thiba, Karima, Kiratina, and Kasarani, while Tebere location had six villages which included Kamucege, Gathigiriri, Block, Kiarukungu, Mahigaini, and Kirogo. Using random sampling, four villages were selected in Thiba location which included Karima, Kasarani, Gakungu, and Rurumi, and three villages in Tebere location including Kirogo, Kamucege, and Block villages.

The study then used probability proportional to size sampling to determine the minimum number of households to be sampled per village. Each study village was mapped, whereby all the households in each village were recorded. Systematic sampling was then used to select households within each village. The first household was randomly selected at the center of the village and, thereafter, every third household from each direction of the first household was surveyed. Household heads or their representatives from the sampled households in each village were interviewed. Sensitization meetings were held in all the villages with the community members, community leadership, and the local administration to explain to them the study and seek their consent.

In each of the sampled households, all individuals present at the time of survey from age 2 years were asked to provide a stool sample after consenting and assenting for the younger children. A written informed consent was further obtained from each participating household head prior to the survey.

### 2.4. Stool Sample Collection and Laboratory Analysis

In each household, data on selected demographic characteristics like age and gender of every participant who consented to provide a stool sample were recorded, and the person was provided with a clean, dry, and leak proof stool container labeled with a unique identifier and asked to provide a stool sample. The samples were then collected in a cooler box and transported to the laboratory for examination. Screening of STH eggs was based on duplicate Kato-Katz thick smears of 41.7 mg prepared from fresh stool samples to determine the prevalence and intensity of STH [36]. For quality assurance, a random sample of 10% of all the positive and negative slides read each day was randomly reexamined by third experienced laboratory technologists.

### 2.5. Ethical Considerations

The study was reviewed and approved by the Scientific and Ethical Review Unit (SERU) of the Kenya Medical Research Institute (KEMRI), number (KEMRI/SERU/ESACIPAC/007/ 3326), and Meru University of Science and Technology Institutional Research Ethics Review Committee (MIRERC), number MIRERC/001/2017. The study received further clearance from Kirinyaga County Health team. A written informed consent (Swahili translated) to participate in the study was obtained from all the adults and an assent sought from all participating children before conducting an interview or collecting a stool sample. For illiterate individuals, a thumb print was used to sign the assent and consent forms after a clear description of the study objectives and acceptance to participate.

### 2.6. Data Management and Analysis

Data collected was entered and stored in an excel spreadsheet. It was then counter-checked for accuracy. Statistical analyses were carried out using STATA version 14.0 (Stata Corporation, College Station, TX, USA). Infection with STH was defined based on the presence of an egg across the duplicate slide readings while intensity was expressed as the arithmetic mean of eggs per gram (epg) of faeces across the two slides. The prevalence of STH was calculated as percentage of the positive participants. The 95% confidence intervals (95% CI) were calculated using binomial logistic regression. Infection intensities were classified into light, moderate, and heavy infections, according to WHO guidelines [37].

## 3. Results

The study enrolled a total of 905 households in seven villages. From the selected households, a total of 3,292 individual members gave a stool sample. Each household had an average of four members. The mean age was 27 years (standard deviation ± 18 years) with an age range of 2 to 98. Out of the total participants, 255 (7.8%) were of preschool going age, 1,101 (33.5%) of school age, and the rest 1.936 (58.8%) of adult age group. Over half of the study participants 1770 (53.8%) were female (Table 1).

### 3.1. Prevalence of STH and Other Helminths among the Study Participants

Of the 3,292 stool samples analysed in this study, only 13 of them tested positive for any STH. Of the 13 samples that tested positive for STH infection, 12 had* Trichuris trichiura* and one had hookworm infection. There was no sample that tested positive for* Ascaris lumbricoides*. Of the 13 STH infections, five were of preschool age (>6 years), seven cases were of school going age (6-18 years), and one was an adult. The single case of hookworm was a male adult of 20 years. Male participants were more infected with STH (61.5%) than female. Other helminths detected in the study included* S. mansoni* 20.1% (95% CI 18.8-21.5%),* Hymenolepis nana* 0.9% (95% CI 0.6 – 1.3%), and one case of Taenia species (Table 2). Analysis of the stool samples also revealed that 672 participants were infected with either species of STH or* S. mansoni *or both (prevalence: 20.4%, (95% CI: 19.1%- 21.8%).

### 3.2. Household Level Prevalence of STH

Analysis of infections at household level is shown in Table 3. Of the 905 households whose members were enrolled in the study, 9 households had at least one member who was positive for STH. Another 431 households had at least one member who was positive for either STH or* S. mansoni* or both (prevalence: 47.6%; 95% CI: 44.4% - 50.9%). The proportion of households which had at least one member testing positive for* S. mansoni *was 47.0% (43.7% - 50.2%). The household level prevalence of* H. nana* was 3.1% (95% CI 2.2% - 4.4%). Hookworm and* T. trichiura* infections were reported in, respectively, one and eight of the nine households whose members were positive for STH.* A. lumbricoides* were not detected in the stool specimens observed in this study.

### 3.3. Intensities of Infections

The intensities of intestinal parasites' infections are displayed in Table 4. The twelve cases of* T. trichiura *infections recorded were of light intensity (1-999 epg). The number of eggs observed for* T. trichiura *ranged from 12 to 72 eggs per gram. The range for the number of eggs detected in samples that were positive for infection with* H. nana* was 408 – 15480 epg while the median (IQR) egg counts was 3,468 (606 – 12,240) epg of faeces. Hook worm had low intensity infection of 24 epg. The number of* S. mansoni *eggs observed in the specimens ranged from 12 to 5,244 eggs per gram (epg) of faeces with a median interquartile range (IQR) of 60 (24 - 156) epg. Majority of the* S. mansoni* infections (65.3%) were of light intensity (1-99 epg).

## 4. Discussion

The main goal of STH control programs, which typically involves preventive chemotherapy, delivered through schools [12], is to reduce the prevalence of moderate to heavy intensity of infections of any STH to below 1% of the at-risk population [38]. Other than the school going children, WHO recommends that other at-risk populations in the community be included in the preventive chemotherapy. In Kenya the control strategy largely is through school based deworming programme, which was launched in the year 2009 with an aim of reducing the prevalence of STH in school age children to below 4% [39, 40].

The current findings show very low infection with STH among all age groups, including preschool (<6 years) and school-aged children (6-18 years) as well as the adult population (>18 years) in an area that has been endemic for these infections. These results support previous findings which have reported low prevalence of STH among preschool children [27] and school age children [33]. In the more than 10 years through which the SBD programme has happened in the study area, the adult community have not received treatment, yet only one adult was found to be infected with hookworm. This supports previous studies which have reported that treating school children through SBD may also benefit the adult age groups even when they do not receive treatment [30]. Previous modeling studies have suggested that school-aged deworming may confer little benefits outside the children being dewormed and that intensifying either treatment coverage or increasing the frequency of treatment may not have an impact on the larger community [31].

The baseline results at the beginning of the SBD programme in Mwea showed that the highest burden of STH was hookworm which was highest among the almost adult age group 16-19 years, where in some schools the prevalence was as high as 28.5%. The prevalence of* Ascaris lumbricoides* and* Trichuris trichiura* was low in all the age groups [21]. In the current study, only one hookworm infection was reported. These results suggest that sustained SBD for a longer period may actually have an impact on the larger community, especially for hookworm species. However, in countries where resources are scarce, sustaining SBD for a longer period may not be a viable option as it is costly and many of such programs are usually donor funded. Therefore incorporating other control strategies to the SBD programs such as improvement in the sanitation conditions both in schools and at home may help countries achieve STH elimination much sooner.

Treating school going children basically reduces the rate at which infective stages of the STH are released to the environment. When this is sustained over a period of time, the entire community may indirectly benefit from the treatment even when they are not treated as there will be reduced exposure to infective eggs and larvae in the environment [30, 41]. Indeed, studies have shown that treating a few individuals in the community can impact on the effective reproductive number of the parasites and therefore reduced exposure to infective stages in those untreated [31]. STH parasites reproduce sexually within the human host; thus, both sexes need to be present within a single host for them to produce fertilized eggs [42] which are expelled to the environment. Treating school age children over a long period of time reduces the chances of both sexes of STH residing in a single human host thus limiting their sexual reproduction. This may explain the low prevalence recorded in the present study after a long period of SBD. In the study area, SBD has been ongoing since the year 2004 [24, 39, 40], with a two-year lapse (2010 and 2011) and then uninterrupted since 2012 to date.

These findings also point to a possibility of the study area reaching a breaking point for all the STH if SBD is sustained. Breaking point typically occurs at levels of below one parasite owing to the aggregated distribution of worm numbers per host [8]. This therefore calls for frequent monitoring and evaluation of the impact of SBD which are not only school based but which involves the entire community. Sustained prevalence and intensity evaluations in this setting will eventually provide useful practical, field based data on how long SBD should be administered to school children for eventual interruption of STH in the whole community. Currently, this information has been provided through mathematical models [8].

Recent studies have suggested the need to shift from morbidity control to interruption of transmission for STH [7, 32], noting that in areas of low transmission, treating school children alone over a long period of time could actually interrupt transmission [43]. Such a shift will require availability of prevalence and intensity data for all age groups and not only the school going children. As the worm burden reduces to such low levels, future surveys need to use diagnostic techniques which are more robust and whose sensitivity is not affected by reduction in worm loads. Kato Katz technique, recommended by WHO for quantification of STH eggs in stool [37] is the most common technique used in field surveys. It is a relatively cheap technique that is easy to apply in resource poor settings [44]. However, its sensitivity has been shown to reduce with the reduction of STH prevalence [45, 46], making it unsuitable in low STH intensity settings. Use of this technique in our study is a potential limitation owing to the low prevalence of STH reported.

The FLOTAC technique has been shown to have high sensitivity even in low intensity settings [45, 47]. It however requires a centrifuge and longer sample preparation time [48]. Future STH evaluations in this area should endeavor to collect multiple consecutive samples to cater for the daily variations of egg excretion and the nonequal distribution of eggs in a stool sample, which leads to varying egg counts between stool samples from the same person [49], and consider use of the FLOTAC technique for stool diagnosis.

## 5. Conclusion

This study shows low prevalence of STH among all age groups including the preschool, school going, and the adult in the community, suggesting that long term SBD may also be benefitting the untreated groups and thus the potential to achieve STH elimination in the area. This study recommends that SBD is continued among school going children so as to sustain the low worm load and probably eventual elimination of STH. The study further recommends that future surveillances should not only be focused on the school age children but also include the larger community.

## Figures and Tables

**Table 1:** Demographic characteristics of the study participants.

Characteristic	Frequency (n=3292)	Percentage (%)
Age (Years)		

<6	255	7.8

6-18	1,101	33.5

>18	1,936	58.8

Gender		

Male	1522	46.2

Female	1770	53.8

Village		

Gakungu	431	13.1

Kamucege	532	16.2

Karima	391	11.8

Kasarani	454	13.7

Kirogo	381	11.8

Rurumi	737	22.3

Block	366	11.1

**Table 2:** Prevalence of STH and other helminths among the study participants.

Species	Number	Prevalence (%)	95% Confidence Interval
STH (n=3292)			

Positive	13	0.4	0.2 - 0.7

Negative	3279	99.6	

*T. trichiura (n=13)*			

Positive	12	92.3	66.7 - 98.6

Negative	1	7.7	

*Hookworm (n=13)*			

Positive	1	7.7	1.4 - 33.3

Negative	12	92.3	

Other Helminths			

*S. mansoni (n=3292)*			

Positive	663	20.1	18.8 - 21.5

Negative	2629	79.9	

*H. nana* (n=3292)			

Positive	29	0.9	0.6 - 1.3

Negative	3263	99.1	

*Taenia* (n=3292)			

Positive	1	<0.1	

Negative	3291		

**Table 3:** Prevalence of STH and other helminths at the household level.

Species	Number	Prevalence (%)	95% Confidence Interval
STH (n=905)		0.0	

Positive	9	1.0	0.5-1.9

Negative	896	99.0	

*T. trichiura (n=9)*			

Positive	8	88.9	56.5 - 98.0

Negative	1	11.1	

*Hookworm (n=9)*			

Positive	1	11.1	2.0 - 43.5

Negative	8	88.9	

Other Parasites			

STH /* S. mansoni (n=905)*			

Positive	431	47.6	44.4-50.9

Negative	474	52.4	

*S. mansoni (n=905)*			

Positive	425	47.0	43.7-50.2

Negative	480	53.0	

*H. nana* (n=905)			

Positive	28	3.1	2.2- 4.4

Negative	877	96.9	

*Taenia* species (n=905)		0.0	

Positive	1	0.1	0.0 - 0.6

Negative	904	99.9%	

**Table 4:** Intensity of STH and other helminths.

Species	Number	%
STH		

*T. trichiura *(n=12)		

Range (Min -max)	12 - 72	

Median (Interquartile range (IQR))	12 (12 – 12)	

Light (1-999 epg)	12	100.0

Moderate (1,000 - 9,999 epg)	0	0.0

Heavy (≥10,000 epg)	0	0.0

Other Helminths		

*S. mansoni *(n=663)		

Range (Min -max)	12 - 5244	

Median (Interquartile range (IQR))	60 (24 - 156)	

Light (1-99 epg)	433	65.3

Moderate (100 - 399 epg)	150	22.6

Heavy (≥400 epg)	80	12.1

*H. nana *(n=29)		

Range (Min -max)	408 - 15480	

Median (Interquartile range (IQR))	12240 (606 - 3468)	

## Data Availability

The data used to support the findings of this study are available from the corresponding author upon request.
